# Clinical practice guideline on the prevention and management of neonatal extravasation injury: a before-and-after study design

**DOI:** 10.1186/s12887-020-02346-9

**Published:** 2020-09-23

**Authors:** Kam Ming Chan, Janita Pak Chun Chau, Kai Chow Choi, Genevieve Po Gee Fung, Wai Wa Lui, Meme Suk Ying Chan, Suzanne Hoi Shan Lo

**Affiliations:** 1 Department of Paediatrics and Adolescent Medicine, United Christian Hospital, Kowloon, Hong Kong; 2 The Nethersole School of Nursing, Faculty of Medicine, The Chinese University of Hong Kong, Shatin, N.T., Hong Kong; 3 Pharmacy Department, United Christian Hospital, Kowloon, Hong Kong

**Keywords:** Intravenous nursing, newborn infant, neonatal intensive care units, evidence-based professional practice

## Abstract

**Background:**

Extravasation injury resulting from intravenous therapies delivered via peripheral intravenous catheters or umbilical and peripherally inserted central venous catheters is a common iatrogenic complication occurring in neonatal intensive care units. This study aimed to evaluate the effectiveness of an evidence-based clinical practice guideline in the prevention and management of neonatal extravasation injury by nurses.

**Methods:**

A controlled before-and-after study was conducted in a neonatal unit. The clinical practice guideline was developed, and a multifaceted educational program was delivered to nurses. Neonatal outcomes, including the rates of peripheral intravenous extravasation and extravasation from a central line, were collected at the pre- and post-intervention periods. Post-intervention data for nurses, including the nurses’ level of knowledge and adherence, were collected at six months after the program.

**Results:**

104 and 109 neonates were recruited in the pre-intervention period (control) and the post-intervention period (intervention), respectively. The extravasation rate before and after the intervention was 14.04 and 2.90 per 1,000 peripheral intravenous catheters days, respectively. The adjusted odds ratio of peripheral intravenous extravasation post-intervention compared with that of pre-intervention was 0.20 (95% confidence interval: 0.05–0.74; *p* = 0.02) after adjusting for peripheral intravenous catheter days. The extravasation from a central line rate of the control and intervention groups post-intervention was 4.94 and zero per 1,000 central venous catheter days, respectively. Fifty-nine registered nurses were recruited. At six months post-program, there were significant improvements in the nurses’ level of knowledge and adherence.

**Conclusions:**

These findings suggest that the implementation of an evidence-based clinical practice guideline significantly reduced the rate of peripheral intravenous extravasation and extravasation from a central line in neonates. However, to maintain nurses’ knowledge and adherence to the evidence-based practice, the educational program will have to be conducted periodically and incorporated into the nurses’ induction program.

**Trial registration:**

ClinicalTrials.gov, Identifiers: NCT04321447. Registered 20 March 2020 - Retrospectively registered.

## Background

Extravasation injury (EI) resulting from intravenous (IV) therapies delivered via peripheral intravenous catheters or umbilical and peripherally inserted central venous catheters (CVCs) is a common iatrogenic complication occurring in neonatal intensive care units (NICUs) [1, 2]. The incidence and prevalence of peripheral intravenous extravasation among neonates is approximately 12.6 per 100 peripheral intravenous catheter days and 38 per 1,000 neonates, respectively [3, 4]. Serious life-threatening cases of peripheral intravenous extravasation and extravasation from a central line have been reported in 92% of the surveyed NICUs in Australia and New Zealand [5].

Studies have reported that neonates born before 37 weeks of pregnancy; having low birth weight, active limb movements, and longer in-situ peripheral intravenous catheter duration; and receiving parenteral nutrition are prone to developing EI [1, 3, 6–8]. The type of peripheral intravenous catheters used and the insertion of disproportionately large peripheral intravenous catheters are also associated with neonatal extravasation [9, 10]. No clinical practice guidelines (CPGs) on the prevention and management of neonatal EI in NICUs have yet been established in Hong Kong. There are also reported incidences of EI caused by the administration of acyclovir and extravasation of parenteral nutrition via umbilical venous catheters in the study neonatal unit in Hong Kong. Therefore, an evidence-based CPG on the prevention and management of neonatal EI and a multifaceted educational program for nurses was developed and evaluated in this study.

The purposes of the study were to (1) evaluate the effectiveness of the evidence-based CPG in the prevention and management of neonatal EI, and (2) examine the change in nurses’ knowledge of and adherence to the prevention and management of neonatal EI after the introduction of the educational program and dissemination of the CPG.

### Hypotheses of the study

It was hypothesized that after completing the educational program and disseminating the CPG, there would be (1) a significant reduction in the incidence of peripheral intravenous extravasation and extravasation from a central line injuries; and (2) a significant improvement in nurses’ knowledge on and practice in the prevention and management of neonatal EI.

## Methods

### Design and settings

A study with controlled before-and-after design was conducted in a neonatal unit in a regional hospital in Hong Kong. It adhered to CONSORT guidelines. Fifty-nine registered nurses were working full-time in the unit. The unit offers 45 beds for neonates who require intensive or special care. Approximately 98% of the neonates in the unit required IV medication or infusion therapy.

### Participants

All nurses and neonates were recruited by convenience sampling. Neonates who were admitted for IV medication or infusion therapy and whose parents were ≥ 18 years old and able to communicate in Chinese or English were recruited. Neonates who were born with congenital dermatological problems such as ichthyosis and epidermolysis bullosa were excluded because of difficulties in determining whether skin tears or blisters were caused by extravasation [11]. Nurses (*n* = 03) who were unable to participate in the knowledge test and audit because of leave arrangements were excluded.

### Sample size estimation

The peripheral intravenous extravasation rate in the study unit was 5.3 per 1,000 peripheral intravenous catheter days) [12]. Using PASS 12 (NCSS, Kaysville, USA), it was estimated that at least 6,970 catheter days would give the study 80% power to detect a 50% reduction in the peripheral intravenous extravasation rate after implementing the CPG at a two-sided 5% significance level. Given a 110% utilization rate of the facility and 98% of the neonates being eligible, an observational period of (6,970 / 45) / 1.1 / 0.98 = 143 IV catheter days before and after implementing the CPG was considered sufficient. Using PASS 12, it was estimated that 52 nurses would give the study 80% power to detect an effect size of 0.4 at a two-sided 5% significance level.

### Intervention

The CPG, including 27 recommendations, was developed by a panel comprising a neonatologist, a clinical pharmacist, a ward manager, a nurse consultant, two advanced practice nurses, and two neonatal nurses after an extensive review of the literature and critical appraisal of the available evidence. Of the recommendations, 11 were about EI prevention and 16 about EI management. The grade of recommendation ranged from A (based on systematic reviews) (four items) to D (based on expert opinion) (nine items) (Refer to Table 5 for details of CPG items). A multifaceted educational program was delivered by a nurse consultant with expertise in neonatal intensive care. The program included (1) computer-assisted instructions on prevention, assessment, and grading of EI severity; (2) a 60-min lecture on the CPG; and (3) simulation training in EI management based on three case scenarios (one about peripheral intravenous extravasation and two about extravasation from a central line).

**Table 1:** Demographic and clinical characteristics of the neonate participants

Variables	Control Group (*n* = 104) *F*(%)	Intervention Group (*n* = 109) *F* (%)	*p* value for difference
**Gender**			0.433^‡^
Male	59 (56.7)	56 (51.4)	
Female	45 (43.3)	53 (48.6)	
**Gestational age (week)**	Mean (SD): 36.80 (4.31)	Mean (SD): 36.47 (4.65)	0.593^#^
**Birth weight (kg)**	Mean (SD): 2.59 (0.95)	Mean (SD): 2.53 (0.92)	0.646^#^
**Level of care**			0.998^‡^
Special care baby unit	62 (59.62)	65 (59.63)	
Neonatal intensive care unit	42 (40.38)	44 (40.37)	
**Maturity**			0.776^‡^
Full term	64 (61.54)	65 (59.63)	
Pre term	40 (38.46)	44 (40.37)	
**Mode of delivery**			0.207^‡^
Spontaneous vaginal	47 (45.19)	40 (36.70)	
Assisted/Instrumental^a^	57 (38.46)	69 (63.30)	
**Cause of hospitalization**			0.962^‡^
Infection	40 (38.46)	40 (36.70)	
Prematurity	32 (30.77)	34 (31.19)	
Other diseases^b^	32 (30.77)	35 (32.11)	
**Apgar score**	Mean (SD):	Mean (SD):	
1 minute	8.82 (1.80)	8.60 (1.81)	0.37^#^
5 minutes	9.30 (1.25)	9.29 (1.04)	0.98^#^
**SNAPPE II score (NICU)**	Mean (SD): 20.86 (22.76)	Mean (SD): 21.93 (17.71)	0.81^#^

*Abbreviations:F* Frequency, *NICU* Neonatal intensive care unit, *SD* Standard deviation, *SNAPPE II* Score for Neonatal Acute Physiology with Perinatal Extension-II

‡ By χ^2^test

# *p* value for mean difference by independent sample *t-*test

^a^ Assisted/Instrumental mode of delivery included caesarean section, vacuum extraction, and assisted breech

^b^ Other diseases included respiratory, cardiovascular, neurological, hematological, gastrointestinal and renal diseases

**Table 2:** Details of the intravenous therapy for the neonate participants

Variables	Control Group (*n* = 104) *F* (%)	Intervention Group (*n* = 109) *F* (%)	*p-*value for difference
Peripheral IV device for continuous infusion.	53 (50.96)	52 (47.71)	0.64^‡^
Peripheral IV device with vesicant administration.	11 (10.58)	17 (15.60)	0.28^‡^
Number of peripheral IV catheter in-situ.	Mean (SD) 2.88 (2.85)	Mean (SD) 2.86 (2.96)	0.96^#^
Peripheral IV catheter days.	Mean (SD) 8.22 (8.76)	Mean (SD) 9.48 (8.79)	0.30^#^
Number of patients with CVC.	26 (25%)	31 (28.44%)	0.40^‡^
Number of CVC in-situ.	Mean (SD) 1.58 (0.50)	Mean (SD) 1.45 (0.51)	0.36^#^
CVC days.	Mean (SD) 15.58 (8.41)	Mean (SD) 14.23 (6.97)	0.51^#^

*Abbreviations:CVC* Central venous catheter, *F* Frequency, *IV* Intravenous,*SD* Standard deviation ‡ By χ^2^test

# *p-*value for mean difference by independent sample *t-*test

### Ethical consideration

This study was approved by the Joint Chinese University of Hong Kong-New Territories East Cluster Clinical Research Ethics Committees of The Chinese University of Hong Kong (CREC Ref. No.: 2015.405) and the Research Ethics Committee (Kowloon Central/ Kowloon East) (Ref.: KC/KE-15-0159/ER-1) and was conducted according to the guidelines laid down in the Declaration of Helsinki. Written informed consent was obtained from the nurses and the parents of all participating neonates.

### Outcome measures

Data were collected at the pre-intervention period (before implementation of the CPG) (an observation period of 143 IV catheter days) and the post-intervention period (a period of 143 IV catheter days starting 1 day after the announcement of implementing the CPG to ensure that all nurse participants, regardless of their shift duties, had completed the program and noted the date of implementation). Post-intervention data for nurses were collected at 6 months after the program. The outcomes included neonatal outcomes and nurse outcomes.

#### Neonatal outcomes

The outcomes included Neonatal outcomes – (1) Peripheral intravenous extravasation rate: the number of peripheral intravenous extravasation incidents divided by the total number of peripheral intravenous catheter days and multiplied by 1,000 and (2) Extravasation from a central line rate: the number of extravasation from a central line incidents divided by the total number of CVC days and multiplied by 1,000 [13]. A neonatal specialty nurse with 17 years of post-registration experience and a neonatologist individually graded the peripheral intravenous extravasation injury using the Pediatric Peripheral Intravenous Infiltration Assessment Tool [14]. Grade 2 to 4 peripheral intravenous extravasation injuries were considered [15]. Any discrepancy in the scores was resolved by a consensus between the nurse and the neonatologist. Injuries resulting from extravasation from a central line were diagnosed by two neonatologists based on radiological and ultrasonic examinations [16].

#### Nurse outcomes

Nurse outcomes **–** (1) Level of knowledge: determined using a self-developed 15-item Neonatal Extravasation Knowledge Test (NEKT) comprising 4-option multiple-choice questions about risk factor identification and prevention and management of EIs; each correct answer is scored 1, and each incorrect answer is scored 0. The total score was obtained by dividing the total number of correct answers by the total number of questions and multiplied by 100. A higher score indicated a higher level of knowledge. The NEKT was previously reviewed by five neonatal experts (content validity index: 0.95) [17]. Thirty ex-neonatal nurses completed the pilot test, and the Kuder–Richardson reliability coefficient was 0.761. (2) Nurses’ adherence: determined using a 27-item audit tool developed by the research team based on the CPG. All items, covering assessment, prevention, and management of EIs, were rated by observation, checking documentation, or asking nurses about the items. The items were scored as “Yes” (satisfactory performance), “No” (unsatisfactory performance), or “NA” (not applicable to the situation). The rate was obtained by dividing the total number of “Yes” items by the total number of items minus the total number of “NA” items and multiplied by 100 (range: 0–100). A higher rate indicated better adherence to the CPG [18]. The tool was reviewed by five neonatal experts (content validity index: 0.97). A pilot test among 25 neonatal nurses showed an intra-class correlation coefficient of 0.95.

#### Neonates’ and nurses’ characteristics

The collected data included the neonate participants’ sex, gestational age, birth weight, health status, mode of delivery, Apgar score, maturity and diagnosis, and Score for Neonatal Acute Physiology with Perinatal Extension-II scores; high risk medications for extravasation, number, and duration of peripheral intravenous catheter and CVCs, types of fluid administration, CVC tip location; peripheral intravenous extravasation and extravasation from a central line injuries and their severity; and the nurse participants’ academic achievement, year of neonatal experience, and professional training in neonatal nursing.

### Statistical analyses

The neonates’ and nurses’ characteristics are presented as frequency and percentage for categorical or nominal variables and as mean and standard deviation for continuous variables. Independent *t*-tests and Pearson’s chi-square tests were performed to compare the mean and proportional differences in neonate outcomes between the pre- and post-intervention periods. Logistic regression was used to estimate the odds ratio of EI in the post-intervention period compared with that in the pre-intervention period with adjustment for catheter days. The NEKT scores and adherence rates in the pre- and post-intervention periods were compared using paired *t*-tests. Subsequent, each question/audit item was analyzed using McNemar tests with Bonferroni correction for multiple comparison performed to determine which areas or questions had been improved. All analyses were performed using IBM SPSS Statistics Version 23 (IBM Corp., Armonk, NY). All statistical tests were two-sided (significance level set at 0.05).

## Results

### Neonatal outcomes

Two independent groups of neonates were recruited before and after the educational program and the implementation of the CPG. Data were collected for 143 days during both before and after the intervention. In total, 104 and 109 neonates recruited in the pre-intervention period (control group) and the post-intervention period (intervention group), respectively. The response rates in the control and intervention groups were 78.8% and 81.3%, respectively. No significant difference was observed in the demographic and clinical characteristics between the control and intervention groups. The demographic and clinical characteristics of neonates in both groups are shown in Table 1. The details of IV therapy for the neonate participants are summarized in the Table 2. No significant proportional differences were observed in the characteristics of peripheral IV therapy between the two groups. There were 855 and 1,033 peripheral intravenous catheter days with 300 and 312 peripheral intravenous catheters in the control and intervention groups, respectively.

**Table 3:** The characteristics of the nurse participants (*n*=53)

Variables	N	%
**Academic achievement**		
Higher diploma	10	18.87
Bachelor degree	30	56.60
Master degree	13	24.53
**Neonatal nursing experience**		
Less than 1 year	15	28.30
1–5 years	18	33.96
More than 5 years	20	37.74
**Specialty education in neonatal nursing**		
Yes	23	43.40
No	30	56.60

Twelve neonate participants (11.54%) in the control group and three (2.75%) in the intervention group were found to have grade 2 to 4 peripheral intravenous extravasation injuries. No recurrence of extravasation injuries in the same neonate participant occurred in either group. The extravasation rate before and after the intervention was 14.04 and 2.90 per 1,000 peripheral intravenous catheter days, respectively. The adjusted odds ratio of having injuries in the post-intervention period compared with that in the pre-intervention period was 0.20 (95% confidence interval: 0.05–0.74; *p* = 0.02) after adjusting for peripheral intravenous catheter days, indicating that the likelihood of injuries in the post-intervention period was reduced by 80%. The findings suggest that the educational program and the implementation of the CPG led to better outcomes in the target population, including a reduction in the peripheral intravenous extravasation rate.

Not all neonates had peripherally inserted CVCs. Therefore, a subgroup analysis was performed for extravasation from a central line. There were 26 and 31 participants with 41 and 45 CVCs in the control and intervention groups, respectively (Table 2). All CVCs were radiologically confirmed to be appropriately positioned. Total CVC days in the control and intervention groups were 405 and 441, respectively.

No significant proportional differences were observed in the characteristics of IV therapy administered via CVCs between the two groups. Two neonates in the control group, but none in the intervention group, had extravasation from a central line. The extravasation from a central line rate in the control and intervention groups was 4.94 and zero per 1,000 CVC days, respectively. However, because of the zero count in the post-intervention period, the odds ratio could not be estimated.

**Table 4:** Comparison of the nurse participants’ correct responses in the Neonatal Extravasation Knowledge Test

Items	Correct response		*p-*value*
	Pre *F* (%)	Post *F* (%)	
**Prevention of Extravasation Injuries**			
1. Confirm correct CVC catheter tip position radiologically immediately after insertion.	41 (77.36)	53 (100)	< 0.001
2. Assess peripheral IV site every 10 minutes when administer high-risk medications.	25 (47.17)	52 (98.11)	< 0.001
3. Assess peripheral IV site every 10 minutes for phenytoin administration.	28 (52.83)	53 (100)	< 0.001
4. Assess peripheral IV device patency prior to medication administration.	45 (84.91)	51 (96.23)	NS
5. Identify possible signs and symptoms of ECL, e.g., sudden cardiorespiratory collapse, respiratory distress, and edema along the CVC tract.	46 (86.79)	53 (100)	NS
6. Recognize ECL presentation, e.g., peritoneal effusion, pericardial effusion and pleural effusion.	44 (83.02)	53 (100)	NS
**Management of Extravasation Injuries**			
1. Administer hyaluronidase for PIVE caused by parenteral nutrition.	15 (28.30)	41 (77.36)	< 0.001
2. Discontinue IV infusion therapy at once on detection of Grade 3 PIVE.	47 (88.68)	49 (92.45)	NS
3. Identify most appropriate nursing interventions for Grade two PIVE: stop infusion, remove peripheral IV catheter, elevate and hourly observation of the affected limb.	22 (41.51)	50 (94.34)	< 0.001
4. Give hyaluronidase on phenytoin related PIVE.	29 (54.72)	49 (92.45)	< 0.001
5. Identify the most appropriate actions when ECL presents with pericardial effusion.	29 (54.72)	52 (98.11)	< 0.001
**Risk Factors for Neonatal Extravasation Injuries**			
1. Administer dopamine via a CVC.	52 (98.11)	53 (100)	NS
2. Identify the physical characteristics of neonates that are prone to extravasation injuries.	45 (84.91)	49 (92.45)	NS
3. Administer acyclovir via a PIVC is at a high risk of PIVE.	33 (62.26)	49 (92.45)	< 0.001
4. Identify high-risk medications for PIVE, e.g., dopamine, dextrose > 12.5%, parenteral nutrition, caffeine citrate.	25 (47.17)	50 (94.34)	< 0.001

*Abbreviations: CVC* Central venous catheter, *ECL* Extravasation from a central line, *F* Frequency, *IV* Intravenous, *PIVC* Peripheral Intravenous Catheter, *PIVE* Peripheral intravenous extravasation, *NS* Not significant

*Comparisons using McNemar test; Level of significance (*p* < 0.003)

### Nurse outcomes

Fifty-six female nurses participated in the study with a response rate of 100%. Fifty-three of these nurses completed the study, and the attrition rate was 5.36%. The characteristics of the nurse participants are summarized in the Table 3.

The nurses’ total mean NEKT scores were 66.04 (SD, 15.92) and 95.22 (SD, 7.55) in the pre- and post-intervention periods, respectively. This increase in the mean score by 29.18at 6 months after the completion of the educational program was statistically significant (*p* < 0.001) [19]. The NEKT contained 15 items. A McNemar test was performed on each item to examine the significant differences in nurses’ knowledge (Table 4). Post-hoc analyses using the Bonferroni correction for multiple comparisons were performed to avoid inflating the Type I error rate. A significant difference (*p* < 0.003) was observed in the number of correct responses in nine items between the pre- and post-intervention NEKT (Table 4). No significant difference was found in the other six items as their correct responses in the pre-intervention assessment were over 83%. In the post-intervention NEKT, 100% correct responses were observed in five items; all other items except one had greater than 90% correct responses

The nurses’ mean adherence rates were 52.48 (SD, 12.20) and 95.73 (SD, 4.84) in the pre- and post-intervention periods, respectively. This increase in the mean adherence rate by 43.25 at 6 months after the completion of the multifaceted educational program was statistically significant (*p* < 0.0

**Table 5:** Nurses’ adherence to the Clinical Practice Guideline before and six months after intervention

Interventions	Adherence (%)		*p*-value*
	Pre- intervention	Post- intervention	
1. Assess signs and symptoms of PIVE.	88.68	100	NS
2. Assess peripheral IV infusion site/s hourly.	100	100	NA
3. Identify high-risk drugs/infusates for PIVE.	66.04	100	<0.001
4. For high-risk drugs/infusates, assess peripheral IV site/s every 10 minutes.	35.85	100	<0.001
Administer high-risk drugs/infusates via a CVC.	83.02	100	NS
5. Administer parenteral nutrition via a CVC.	96.23	100	NS
6. Assess patency of peripheral IV device/s prior to drug administration.	100	100	NA
7. Secure peripheral IV device with transparent dressing.	100	100	NA
8. Splint extremities with a peripheral IV device in situ.	100	100	NA
9. Assess patient for ECL.	15.09	71.70	<0.001
10. Confirm correct position of CVC tip.	69.81	100	<0.001
11. Initiate actions for managing PIVE at once.	98.11	100	NS
12. Grade PIVE severity with infiltration scale.	0	96.23	<0.001
13. Implement management of Grade 1 and 2 PIVE.	84.91	98.11	NS
14. Notify physician for Grade 3 and 4 PIVE.	66.04	100	<0.001
15. Administer Hyaluronidase for PIVE caused by non-vasopressor drugs/infusates.	39.62	100	<0.001
16. Administer Phentolamine for PIVE caused by vasopressor drugs/infusates.	1.89	90.57	<0.001
17. Initiate non-pharmacological pain relief for extravasation injuries.	77.36	100	<0.001
18. Offer pharmacological pain relief for extravasation injuries.	79.25	100	0.001
19. Observe PIVE with intact skin hourly for 12 hours.	5.66	90.57	<0.001
20. Remove the CVC for ECL.	77.36	81.13	NS
21. Initiate nursing interventions to manage ECL.	16.98	81.13	<0.001
22. Consult wound nurse for wound management.	11.32	90.57	<0.001
23. Document PIVE or ECL on extravasation record.	0	100	<0.001
24. Report extravasation injury to parents.	7.55	96.23	<0.001
25. Update progress of extravasation injury to parents.	1.89	98.11	<0.001
26. Incident reporting.	0	90.57	<0.001

*Abbreviations:CVC* Central venous catheter, *ECL* Extravasation from a central line, *IV* Intravenous, *NA* Not applicable, *NS* Not significant, *PIVE* Peripheral intravenous extravasation

*Comparisons of nominal data: McNemar test; Level of significance (*p *< 0.002)

The McNemar test was performed to analyze the change in the adherence rate for each item, and the results in the pre- and post-intervention audits are shown in Table 5. In the pre-intervention period, the adherence rate (satisfactory performance) ranged from 15.2–100% for items related to EI prevention and from 0–100% for items related to EI management. In the post-intervention period, the adherence rate ranged from 71.70–100%. Post-hoc analyses using the Bonferroni correction for multiple comparisons revealed significant differences (*p* < 0.002) in the adherence rate for 17 items of the audit tool. No significant difference was observed in the adherence rate for six items between the pre- and post-intervention audits. The adherence rate for five items was higher than 80% in the pre-intervention audit, and that for four items was 100% in both the pre- and post-intervention audits (Table 5).

Furthermore, in the pre-intervention audit, 12 items showed adherence rates lower than 50%. Three items with adherence rates of 0% in the pre-intervention audit showed higher than 90% adherence rates in the post-intervention audit. In the post-intervention audit, the adherence rate for 16 items was 100%. The lowest adherence rate in the post-intervention audit was 71.70% observed for the item assessment of extravasation from a central line (Table 5).

## Discussion

A before-and-after study design was adopted as the intervention had to be conducted in the same study unit, which made randomization impossible. A comparison of the neonate participants’ demographic and clinical characteristics and the nature of the IV therapy between the control and intervention groups revealed no significant inter-group differences, indicating homogeneity between the two groups. With the completion of the educational program and the implementation of the CPG, there were decreases in the total numbers of peripheral intravenous extravasation and extravasation from a central line injuries and a reduction in the rate of such injuries. The reduction in the peripheral intravenous extravasation rate was more than four-fold, from 14.04 to 2.90 per 1,000 peripheral intravenous catheter days. The same trend was also found in the rate of extravasation from a central line—a decrease from 4.94 to zero per 1,000 CVC catheter days. The risk of injury was also assessed using logistic regression after adjusting for the difference in peripheral intravenous catheter days between the control and intervention groups. The adjusted odds ratio for peripheral intravenous extravasation injuries during the post-intervention period was 0.20—a statistically significant result that indicated an 80% reduction in the likelihood of developing a peripheral intravenous extravasation injury during the post-intervention period compared with the pre-intervention period. In other words, the intervention effectively reduced the incidence of neonatal peripheral intravenous extravasation injuries. However, as the rate of extravasation from a central line injuries in the intervention group was zero, we could not calculate the risk of injury using logistic regression. The findings suggest that the use of a multifaceted educational program and the implementation of an evidence-based CPG led to better clinical outcomes, including a reduction in the number and rate of both peripheral intravenous extravasation and extravasation from a central line injuries. This marked improvement demonstrated that the neonates clearly benefited from the intervention.

There was a significant improvement in the nurses’ knowledge and practice at 6 months after the completion of the educational program. The average increase in the NEKT score was 29.18 points. The adherence rate in the intervention group increased by an average of 43.26%. In the pre-intervention NEKT, four items had a correct response rate of less than 50%. These four items were the properties of hyaluronidase (28.30%), identification of high-risk medications (47.17%), frequency of assessment of peripheral IV administration of high-risk medication (47.17%), and management of grade 2 peripheral intravenous extravasation injuries (41.51%). These four items were also associated with the greatest improvement in the post-intervention NEKT. Except for identifying the properties of hyaluronidase, the other three items showed an increase in correct responses to > 90% in the post-intervention NEKT. These results indicated that the nurses’ knowledge on the administration and appropriate use of hyaluronidase need to be improved. Two questions were related to the frequency of assessment when high-risk medications are administered via a peripheral IV device. The correct response rates for the two questions differed because one nurse participant did not attempt one of the questions, resulting in a discrepancy. Among the 15 multiple-choice questions, 9 were associated with statistically significant post-intervention knowledge improvements.

In terms of the results of the 27-item audit tool, no statistically significant differences were found between the pre- and post-intervention adherence rates for 10 audit items. Four of the items received 100% adherence rates in the pre-intervention audit. All these four items addressed the practical prevention of peripheral intravenous extravasation: the frequency of assessment of peripheral IV sites, assessment of peripheral intravenous catheter patency before medication administration, securing of peripheral IV devices, and limb immobilization.

During the pre-intervention audit, 12 items scored adherence rates of less than 50%, 3 of which even received zero adherence. These three items were the grading of peripheral intravenous extravasation injuries using an infiltration scale, documentation of peripheral intravenous extravasation on the extravasation record, and incident reporting during mortality and morbidity meetings related to advanced peripheral intravenous extravasation or extravasation from a central line. Before the implementation, the Pediatric Peripheral Intravenous Infiltration Assessment Tool and extravasation record had not been introduced to nurses in the unit, thus resulting in an adherence rate of zero [14]. In contrast, after delivering the multifaceted educational program, all but one item – assessment of extravasation from a central line – received an adherence rate of higher than 80%. Assessment of extravasation from a central line was the only item with an adherence rate of lower than 80% in the post-intervention audit due to incomplete assessment. As such, a gap between the actual nursing practices and evidence-based recommendations concerning the assessment of extravasation from a central line was found. This area of practice warrants attention and reinforcement because although extravasation from a central line is rare, it has detrimental effects. This area could be strengthened through discussions in nursing focus-group meetings, clinical supervision, and feedback from audits. Additional strategies such as development of more case scenarios in the educational package, use of posters, and videos to reinforce the previously taught nursing skills would be helpful to improve nurses’ adherence to evidence-based recommendations on the assessment of extravasation from a central line.

### Implications for practice

EI is a complicated phenomenon characterized by diverse symptom patterns and physical distress ranging from mild erythema to skin necrosis or permanent contracture [20]. This condition is devastating to neonates. With the implementation of the CPG, the rate of grade 2 to 4 peripheral intravenous extravasation injuries decreased. The study results also demonstrated marked improvements in the nurses’ levels of both knowledge and practice. This program is worthy of continuation and should be incorporated into the orientation program for new nurses. Clinical competency is pivotal to the provision of safe patient care, and education is recognized as a route for competence development [21]. In this study, the nurses’ practices improved after the educational intervention. However, over time, both knowledge and practices will change. For sustainability, the educational program and audit will have to be conducted at least annually with organizational support [22].

### Limitations

There has been limited high quality research evidence to inform the development of the CPG on prevention and management of neonatal EI. Further update in the CPG based on the latest available research evidence is needed to inform practice. Both the pre- and post-intervention audits were conducted by a research nurse, and the nurse participants were aware that they were being observed. Therefore, the Hawthorne effect could not be eliminated. To minimize the bias, the research nurse was trained to observe participants’ practice with a non-judgmental and unbiased attitude. Furthermore, the nurses’ satisfaction was not surveyed, despite their significant involvement in the implementation of the evidence-based CPG. The nurses’ satisfaction, feedback, and opinions regarding the implementation process should be explored before introducing another quality improvement project.

## Conclusions

This evaluation study showed that the implementation of an evidence-based CPG significantly reduced the rate of peripheral intravenous extravasation and extravasation from a central line in neonates. The delivery of a multifaceted educational program for the improvement of nurses’ knowledge and practice was a prerequisite for successful implementation. However, to maintain continual improvement in nurses’ knowledge and adherence to the evidence-based practice, the educational program will have to be conducted periodically and incorporated into the nurses’ induction program.

## Abbreviations

CVC: Central Venous Catheter
CPG: Clinical Practice Guideline
EI: Extravasation Injuries
IV: Intravenous
NEKT: Neonatal Extravasation Knowledge Test
NICU: Neonatal Intensive Care Unit

## Availability of data and materials

The datasets used and/or analyzed during the current study are available from the corresponding author on reasonable request.
